# A benchmark of methods for surgical instrument segmentation in endoscopic pituitary surgery

**DOI:** 10.1007/s11548-026-03668-2

**Published:** 2026-05-21

**Authors:** Kendall Feeny, Anjana Wijekoon, Wenhua Wei, Danyal Zaman Khan, Danail Stoyanov, Hani J. Marcus, Sophia Bano

**Affiliations:** 1 Institute of Health Informatics, University College London, London, UK; 2 Department of Computer Science, University College London, London, UK; 3 UCL Hawkes Institute, University College London, London, UK; 4 Department of Neurosurgery, National Hospital for Neurology and Neurosurgery, London, UK

**Keywords:** Medical instrument segmentation, Semantic segmentation, Pituitary surgery, Benchmark

## Abstract

****Purpose**:**

The development of impactful AI solutions for minimally invasive surgeries is reliant on fundamental tasks such as surgical instrument segmentation. The fine-grained information provided can support downstream tasks such as skill assessment, intra-operative decision support, and outcome prediction. However, many procedures which stand to benefit significantly from these applications are under-represented in literature as a result of the heavy requirements for data collection and annotation.

****Methods**:**

This study examines an in-house labelled surgical instrument dataset for endoscopic transsphenoidal pituitary surgery and compares the performance of a number of established segmentation models, with both transformer-based and convolutional architectures.

****Results**:**

Our results show that the highest performance was achieved by the EoMT model, with a DSC of 0.7420 while maintaining a more balanced performance across minority classes. Additionally, our results show the challenges caused by the use of real surgical footage, with highly variable frame contents, and significant class imbalances. This is reflected in the reduced performance across classes with low frequencies which are similar in appearance to more popular classes.

****Conclusion**:**

These findings highlight model architectures with potential for development and application to surgical instrument segmentation in pituitary surgery and additionally emphasise the need for larger datasets and further work to include under-represented surgeries with novel challenges.

## Introduction

The endoscopic transsphenoidal approach (eTSA) is the gold standard for the removal of pituitary adenomas—tumours which form on the anterior pituitary gland at the base of the brain [1]. This minimally invasive surgical approach presents unique and substantial challenges caused by the locality of critical structures, limited space provided by the access via nasal corridor, and as a result exhibits a steep learning curve and challenging outcome prediction. Techniques to address these rely on a fundamental understanding of the procedure through surgical workflow analysis (SWA), ranging from high-level information such as phase recognition to frame-wise semantic instrument segmentation (SIS) [2]. Current pituitary semantic segmentation research has been limited to anatomical segmentation [3].

**Fig. 1:**
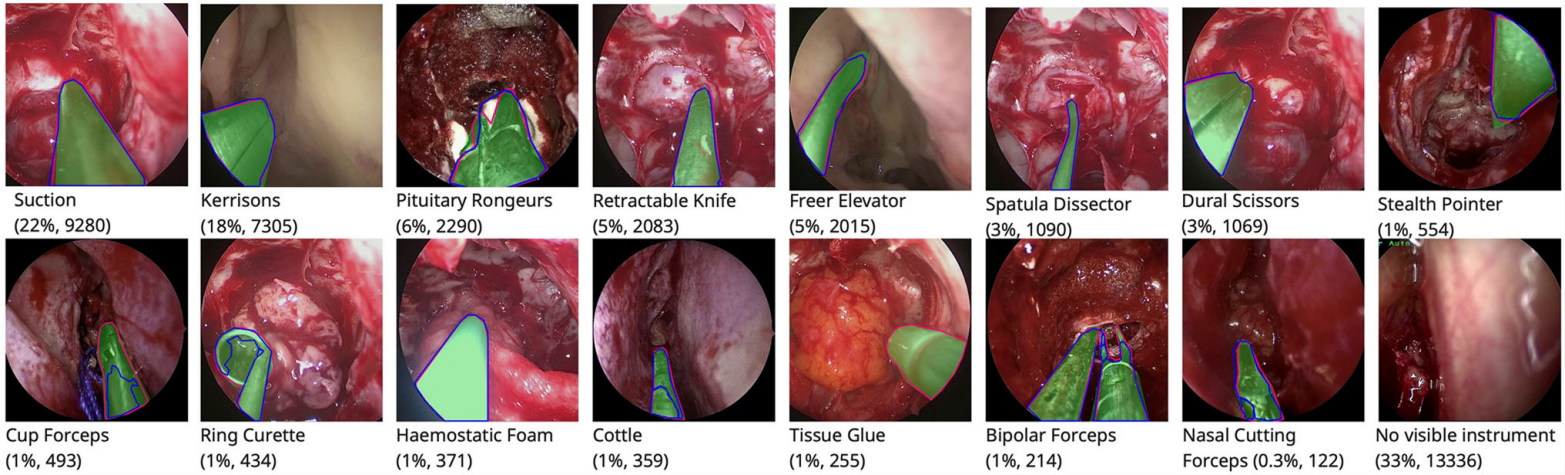
Sample images of all instrument classes with ground truth annotation masks (green), and contour outlines from EoMT (red), and EndoViT (blue)

This work presents the first benchmark of established multi-class SIS models, using an in-patient dataset from eTSA pituitary surgery. The featured neurosurgical instruments are smaller and visually distinct from those seen in many publicly available datasets [4]. The performance of six models, spanning convolutional and transformer-based architectures, is compared, identifying challenges and opportunities for the future development of instrument segmentation methods in endoscopic pituitary surgery, which may also be seen in other under-represented procedures.

## Methods

**Dataset** The dataset used for this study is detailed in Khan et al. [2], consisting of 50 videos of eTSA pituitary surgeries; 120 frames were taken from the beginning of each occurrence of each previously defined step, excluding frames with entire view obscuration by motion blur, smoke or fluid, frames in prolonged periods of inactivity, and frames featuring instruments seen in fewer than 5 videos. Each frame was then annotated by an individual and reviewed by a senior clinical team member, with disagreements resolved through group discussion. The resulting dataset is composed of 37,547 frames with 27,934 instrument masks from 15 instrument classes. The annotations show a substantial class imbalance indicated in Fig. 1, reflecting the intra- and inter-operative variation as surgeons with individual tool and technique preferences work through a complex, multi-stage procedure.

**Models** We train a selection of convolutional and transformer-based semantic segmentation architectures chosen for their prevalence, performance, and availability. U-Net is a well-established, encoder–decoder architecture which remains pervasive in segmentation tasks because of its simplicity, efficacy, and adaptability [5]. HRNet builds on U-Net to produce high-resolution masks from the aggregation of multi-scaled convolutional pathways [6]. DeepLabV3+ targets high-resolution masks with low computational requirements through the spatial pyramid pooling of atrous convolutions [7]. The Vision Transformer (ViT) is a foundational model pre-trained on image-based mask reconstruction. ViT tokenises images and leverages local and global attentions for reconstruction, before being fine-tuned for downstream tasks [8]. EndoViT is a ViT network pre-trained on over 700,000 endoscopic images [9]. The model outperforms task-specific models in surgical action recognition, semantic instrument segmentation, and other SWA tasks. Segformer is a lightweight transformer encoder network that utilises a lightweight MLP decoder, reducing computational requirements through the production of multi-scale encodings inplace of positional encoding [10]. The resulting network achieves comparable performance to other known models with a simpler and more parameter efficient network. Finally, the Encoder-only Mask Transformer (EoMT) adapts the ViT to remove the reliance of task-specific components [11]. EoMT adds learnable queries to processed tokens within the model and uses a mask prediction module in place of a task-specific decoder, eliminating the need for heavy up-sampling.

**Fig. 2:**
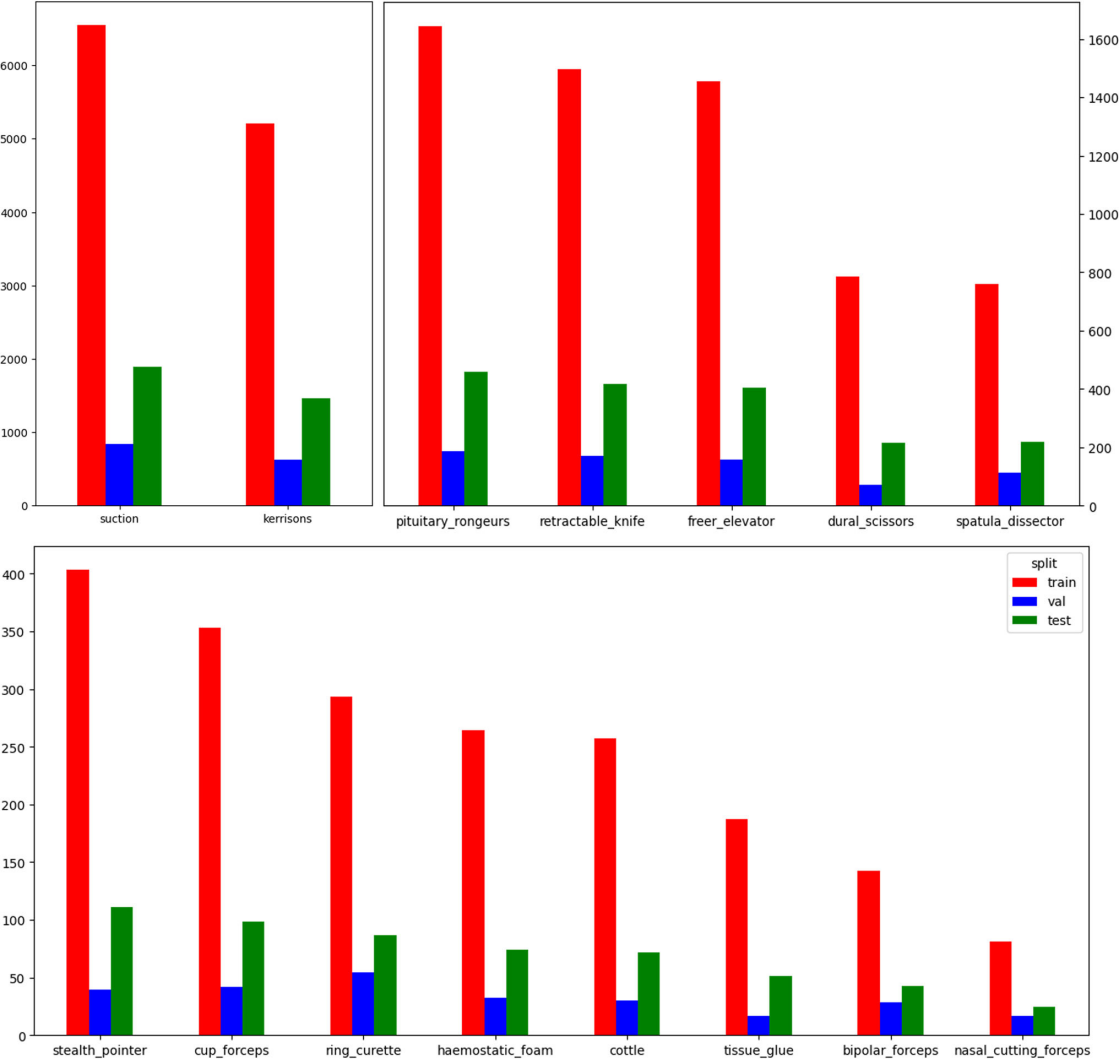
Average class distribution across splits, arranged from most frequent classes (top-left) to least frequent classes (bottom)

**Experimental Setup** To account for substantial class imbalances and inter-video variation a fivefold cross-validation is employed, stratified to maximise minority class presence across folds. The resulting splits are employed per video; each fold composed of frames from 36 training, 4 validation, and 10 test videos as shown in Fig. 2. Where possible, model training methods were extracted from the literature, any not explicitly depicted were tuned on a subset of the training dataset. U-Net weights were initialised as outlined in the literature. HRNet, Segformer, and DeepLabV3+ were all pre-trained using ImageNet. EndoViT was pre-trained on surgical video data for mask reconstruction [9], and EoMT uses the DINOv2 pre-trained weights trained on LVD-142 M (natural images) to initialise the ViT encoder. EndoViT was fine-tuned using a combination of IoU, Dice, and Per-Pixel Accuracy losses. EoMT fine-tuning used a weighted combination of Dice, Per-Pixel Accuracy, and cross-entropy losses. All remaining models were trained on a combination of cross-entropy and Dice loss. Performance is measured utilising the Dice-Sørensen coefficient. Experiments were run on an NVIDIA RTX A6000 GPU (48GB), utilising the PyTorch framework.

## Results and conclusion

As shown in Table 1, class-wise performance was highly varied, with the most frequent classes largely achieving the best performance. This excludes two less frequent but visually distinct classes—haemostatic foam and tissue glue. Convolutional architectures were the worst performing, with several classes scoring 0—this could be attributed to lack of global context or spatial information lost through downsampling. EoMT achieved the highest and most balanced performance, showing promise for application at a substantial computational cost with 315 M trainable parameters, the next largest being EndoViT with 111 M.

**Table 1:** K-fold cross-validation results—*(ordered by frequency)*

Class	U-Net	HRNet	DLV3+	Segformer	EndoViT	EoMT
	[5]	[6]	[7]	[10]	[9]	[11]
*Suction*	0.3294	0.3918	0.2540	0.4059	0.5660	0.8796
*Kerrisons*	0.5088	0.4898	0.3396	0.3343	0.6544	0.9005
*Pituitary rongeurs*	0.3506	0.3729	0.1683	0.3553	0.5145	0.8160
*Retractable knife*	0.4635	0.6016	0.2151	0.2445	0.4741	0.8762
*Freer elevator*	0.2522	0.6082	0.4408	0.6109	0.6252	0.8350
*Spatula dissector*	0.0133	0.0000	0.1911	0.2296	0.2041	0.6634
*Dural scissors*	0.0000	0.0834	0.1444	0.4141	0.5003	0.7857
*Stealth pointer*	0.0302	0.1059	0.2755	0.2092	0.3477	0.7293
*Cup forceps*	0.0000	0.0000	0.0141	0.0642	0.1431	0.4777
*Ring curette*	0.0000	0.0000	0.1617	0.3774	0.2598	0.6965
*Haemostatic foam*	0.5734	0.5863	0.7008	0.7078	0.7507	0.9449
*Cottle*	0.0000	0.0000	0.0549	0.0547	0.2309	0.5090
*Tissue glue*	0.1139	0.0000	0.0950	0.6189	0.5815	0.7648
*Bipolar forceps*	0.0000	0.0000	0.0211	0.0169	0.1643	0.6666
*Nasal cutting forceps*	0.0000	0.0000	0.0000	0.0000	0.0707	0.3293
Mean	0.2243	0.2646	0.2529	0.3517	0.4425	0.7420

In this study, we benchmarked six established models on a new pituitary instrument segmentation dataset. Transformer architectures comprehensively outperformed convolutional ones, emphasising the importance of global context and spatial information. All models achieved reduced performance in classes with fewer occurrences and less distinct appearances, highlighting the potential for future work with added context through techniques such as temporal modelling, domain-specific foundational models, and dataset expansion to include more instances of minority classes.
